# Role of *Akkermansia* in Human Diseases: From Causation to Therapeutic Properties

**DOI:** 10.3390/nu15081815

**Published:** 2023-04-08

**Authors:** Antonio Pellegrino, Gaetano Coppola, Francesco Santopaolo, Antonio Gasbarrini, Francesca Romana Ponziani

**Affiliations:** 1 Internal Medicine and Gastroenterology-Hepatology Unit, Fondazione Policlinico Universitario Agostino, Gemelli IRCCS, 00168 Rome, Italy; 2 Dipartimento Universitario di Medicina e Chirurgia Traslazionale, Università Cattolica del Sacro Cuore, 00168 Rome, Italy

**Keywords:** *Akkermansia muciniphila*, gut, IBD, insulin sensitivity, atherosclerosis, cancer therapy

## Abstract

The gut microbiota plays a critical role in the modulation of host metabolism and immune response, and its impairment has been implicated in many gastrointestinal and extraintestinal diseases. Current evidence shows the well-documented role of *A. muciniphila* in maintaining the integrity of the intestinal barrier, modulating the host immune response, and improving several metabolic pathways, making it a key element in the pathogenesis of several human diseases. In this scenario, *A. muciniphila* is the most promising next-generation probiotic and one of the first microbial species suitable for specific clinical use when compared with traditional probiotics. Further studies are needed to provide more accurate insight into its mechanisms of action and to better elucidate its properties in several major areas, paving the way for a more integrated and personalized therapeutic approach that finally makes the most of our knowledge of the gut microbiota.

## 1. Introduction

In 2004, Muriel Derrien isolated for the first time a bacterium capable of growing on a viscous substrate, such as mucin, and using it as its sole source of energy; as a tribute to the Dutch microbiologist Antoon DL Akkermans and given its ability to grow on a mucin substrate, this bacterium was named *Akkermansia muciniphila*. *A. muciniphila* represents approximately 1–3% of the total gut microbiota in healthy people; it is a non-motile, Gram-negative, non-spore-forming, oval-shaped bacterium belonging to the *Verrucomicrobia* phylum, and it is the first and only member of the phylum *Verrucomicrobia* found in the human gut [1,2,3]. *A. muciniphila* was originally considered a strict anaerobe, but it was recently proved to tolerate small amounts of oxygen and was therefore reclassified as an aerotolerant anaerobe [4].One of the distinguishing features of *A. muciniphila* is its ability to degrade intestinal mucin glycoproteins via enzymes, such as glycosyl hydrolases, proteases, sulphatases, and sialidase, and to use them as a sole source of carbon and nitrogen; this process leads to the production of the short-chain fatty acids (SCFAs) acetate, propionate, and 1,2-propanediol, as well as succinate and sulfate. Due to this process of degradation, *A. muciniphila* promotes mucin turnover and thickening, thereby reinforcing the intestinal barrier and reducing gut permeability to microbial products. A further barrier-reinforcing mechanism is the *A. muciniphila*-induced production of antimicrobial peptides from Paneth cells. SCFAs derived from gut mucin glycoproteins are absorbed in the colon and serve as an energy source for colonocytes, inducing regulatory T cells and exerting anti-inflammatory effects [4,5,6,7,8].

SCFAs are subsequently used by other bacteria in the gut microbial community, such as *Anaerostipes caccae*, *Anaerobutyricum hallii*, and *Faecalibacterium prausnitzii*, to further produce butyrate and propionate [1,6,9,10,11].

In addition to metabolites, the effects of *A. muciniphila* are mediated by exposed active molecules; among these, Amuc_1100 (an outer membrane protein involved in pili formation) can replicate almost all of the effects of live *A. muciniphila* through Toll-like receptor 2 (TLR2) sensing [12,13,14]. TLRs are expressed by a wide range of immune, epithelial, and endothelial cells whose main role is the recognition of microbial structures, capable of stimulating pro- and anti-inflammatory responses with further implications in the regulation of host metabolism [14,15]. The heat stability of these proteins explains why *A. muciniphila* retains most of its effects even after pasteurization (Table 1). In 2021, the safety of pasteurized *A. muciniphila* was positively assessed by the European Food Safety Authority (EFSA) and the Panel on Nutrition, Novel Foods and Food Allergens (NDA) [16], and its production represents the beginning of the new generation of probiotics [17].

**Figure 1:**
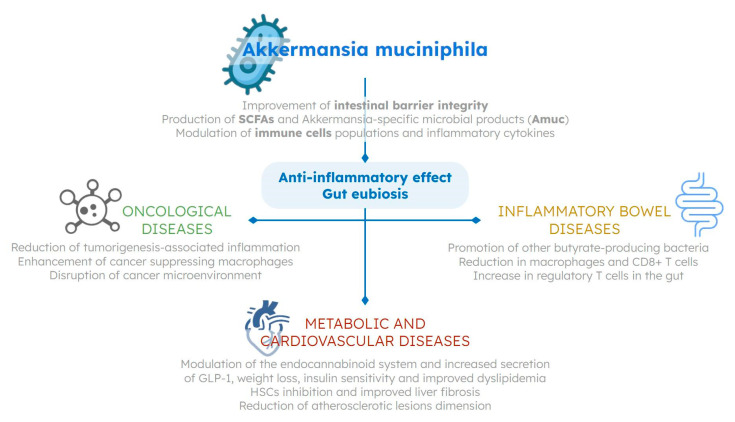
Potential beneficial mechanisms of *Akkermansia muciniphila* in humans. Through the direct and indirect regulation of immune system response, inflammatory, and endocrine pathways, as well as its substantial influence on gut ecology, *Akkermansia muciniphila* has been proven to have a beneficial role in modulating intestinal and extraintestinal diseases. HSCs—hepatic stellate cells; GLP-1—glucagon-like peptide-1; SCFAs—short-chain fatty acids.

Due to its many beneficial effects (Figure 1), it is not surprising that *A. muciniphila* can be used as a biomarker of a healthy host metabolic profile, and that its depletion represents a signature of intestinal dysbiosis across different gastrointestinal and extraintestinal diseases. A reduced abundance of *A. muciniphila* in the gut microbial community has been related to several metabolic and inflammatory diseases, such as obesity, type 2 diabetes, and inflammatory bowel disease; conversely, the administration of live *A. muciniphila* has also shown a protective role in the pathogenesis of cardiovascular disease in mice [18,19,20,21] (Table 2).

This review aims to analyze the mechanisms by which *A. municiphila* is involved in the development and progression of human diseases, with a focus on its potential therapeutic use as a novel probiotic.

**Table 1:** Studies reporting on the beneficial effects of the administration of pasteurized *A. muciniphila* or its components.

Author	Setting	Product Administered	Results	Mechanism of Action
Chelakkot C. et al. 2018 [13]	HFD-fed mice	Akk-EVs	Reduced body weight; Improved glucose tolerance.	Reduced gut permeability with increased tissue expression of tight junction proteins in an AMPK-dependent manner.
Plovier H. et al. 2017 [14]	HFD-fed mice	Live and pasteurized *A. muciniphila* and the outer membrane protein Amuc_1100	Reduced body and fat mass, insulin resistance, and lipid levels (>in mice treated with the pasteurized bacterium or with Amuc_1100).	Enhanced gut barrier function. Amuc-mediated TLR2 activation.
Wang L. et al. 2020 [22]	Mice with DSS-induced colitis and CAC	Pasteurized *A. muciniphila* or the outer membrane protein Amuc_1110	Amelioration of colitis symptoms; improvement of histologic damage; Improvement of CAC symptoms; delayed tumor development; decreased number and area of tumor lesions.	Reduction in macrophage and CD8+ cytotoxic T lymphocyte levels in the colon of mice with DSS-induced colitis; Reduction in markers of DNA damage; cell apoptosis; abnormal proliferation of colonic epithelial cells; Expansion of cytotoxic T-lymphocytes in the colon and mesenteric lymph nodes; modulation of macrophage subpopulations in CAC mice.
Qian K. et al. 2022 [23]	Mice with DSS-induced colitis	Amuc_2109 (a β-acetylaminohexosidase secreted by *A. muciniphila*)	Amelioration of colitis symptoms.	Reduced expression of pro-inflammatory cytokines; Enhanced gut barrier function; Reshaped gut microbiota.
Meng X. et al. 2020 [24]	LS174T cancer cells	Amuc_1434 (a recombinant enzyme derived from *A. muciniphila* able to degrade Muc2)	Inhibition of proliferation and enhanced apoptosis of LS174T cells in vitro.	Enhanced expression of p53, resulting in blockade of G0/G1 cell cycle phase; Enhanced TRAIL-mediated apoptosis pathway.
Luo Z. et al. 2021 [25]	PCa-bearing mice	Akk-Evs	Reduced tumor burden.	Increased infiltration of GZMB+, IFN-γ+ CD8+ lymphocytes, and M1 macrophages in tumor tissue.
Ashrafian F. et al. 2019 [26]	HFD-fed mice	Live *A. muciniphila* + EVs	Reduced body weight and fat mass weight (more significant in Akk-EVs treated mice); Amelioration of lipid profile and glucose levels.	Increased fatty acid oxidation in white adipose tissue; Reduced inflammation in white adipose tissue; Enhanced gut barrier function.
Yang M. et al. 2020 [27]	HFD-fed mice	Three strains of pasteurized *A. muciniphila* with anti-lipogenic activity in vitro	Reduced body weight and fat mass; Improved glucose homeostasis and insulin sensitivity; Prevention of liver steatosis, reduction of liver injury.	Reduced expression of lipogenic-adipogenic markers (as PPARγ) in adipose tissue and liver; Increased gut production of GLP-1 and PYY; Increased expression of IRS-1; reduced expression of leptin gene in adipose tissue; Inhibition of low-grade intestinal inflammation, restoration of damaged gut integrity.
Depommier C. et al. 2020 [28]	HFD-fed mice	Pasteurized *A. muciniphila*	Reduced body and fat mass weight.	Reduced expression of perilipin-2 (a protein involved in the regulation of lipolysis) in adipose tissues; Reduced expression of gut GLUT2, GLUT5, and SGLT1 with consequent decrease in carbohydrate absorption.
Depommier C. et al. 2019 [29]	Overweight/obese insulin-resistant patients	Live and pasteurized *A. muciniphila*	Both preparations were safe and well-tolerated; Pasteurized *A. muciniphila* supplementation significantly improved insulin sensitivity and reduced insulinemia and plasma total cholesterol; Pasteurized *A. muciniphila* supplementation slightly decreased body weight, fat mass, and hip circumference.	
Zhang L. et al. 2018 [30]	Streptozotocin-induced diabetic rats	Live and pasteurized *A. muciniphila*	Both preparations significantly increased blood concentration of HDL and decreased hepatic glycogen, serum PAI-1, TNF-α, LPS, malondialdehyde, and total GLP-1, thereby ameliorating the course of the disease.	
Raftar S. et al. 2020 [31]	Quiescent and LPS-activated HSC and HFD-fed mice treated with CCl4	Live and pasteurized *A. muciniphila* and its EVs	Reduced expression of fibrosis markers via activated HSC; Amelioration of liver biochemistry; Attenuation of liver histopathological damage; Reduced expression of fibrosis and inflammatory biomarkers in hepatic tissue.	Reduced expression of TLR2 and TLR4 in HSC; Enhanced gut barrier function; Reshaped gut microbiota.

Abbreviations: HFD—high-fat diet; Akk-EVs—A. muciniphila-derived Extracellular Vesicles; AMPK—AMP-activated protein kinase; TLR2—Toll-like Receptor 2; DSS—dextran sulfate sodium; CAC—colitis-associated cancer; Muc2—mucin 2; p53; tumor protein 53; TRAIL—tumor-necrosis-factor-related apoptosis-inducing ligand; PCa—Prostate Cancer; GZMB+—granzyme B-positive; IFN-γ+—interferon γ-positive; PPARγ—peroxisomes proliferator-activated receptor gamma; GLP-1—Glucagon-like peptide 1; PYY—peptide YY; IRS-1—Insulin Receptor Substrate 1—GLUT2—glucose transporter 2; GLUT5—glucose transporter 5; SGLT1—sodium-glucose transporter 1; HDL—high-density lipoprotein—PAI-1—plasminogen activator inhibitor-1; TNF-α—tumor necrosis factor-α; LPS—lipopolysaccharide; HSC—hepatic stellate cells—CCL4—carbon tetra chloride; TLR4—Toll-like receptor 4.

**Table 2:** Studies reporting on the beneficial effects of the administration of live *A. muciniphila*.

Author	Setting	Product Administered	Results	Mechanism of Action
Everard A. et al. 2013 [5]	HFD-fed mice	Prebiotics, live, and heat-killed *A. muciniphila*	Prebiotic feeding restored gut levels of *A. muciniphila* and reversed HFD-induced metabolic changes; Viable but not heat-killed *A. muciniphila* administration reduced body weight, fat mass, fasting, post-prandial hyperglycemia, and insulin resistance in HFD-fed mice.	Reduction in hepatic glucose-6-phosphatase expression (thereby suggesting a reduction in gluconeogenesis); Increased expression of markers of adipocyte differentiation and lipid oxidation without affecting lipogenesis markers; Increased Endocannabinoid production in the ileum; Enhanced gut barrier function.
Li J. et al. 2016 [21]	Apoe (−/−) mice on Western diet	Live *A. muciniphila*	Reduced formation of atherosclerotic lesions; Reduced inflammation.	Reduced gut permeability and metabolic endotoxemia.
Zhang T. et al. 2020 [32]	UC and CD patients with active disease despite conventional therapy	WMT	After WMT, 53.7% of patients achieved clinical response with a significantly increased colonization rate of *Akkermansia* compared with pre-WMT; Positive correlation between patients and donors in the abundance of *Akkermansia* after WMT.	
Zhai R. et al. 2019 [33]	Mice with DSS-induced colitis	Live *A. muciniphila*	Improved colitis-related clinical parameters including spleen weight, colon inflammation index, and colon histological score; Downregulation of pro-inflammatory cytokines in the colon.	Increased conversion of CD4+ T cells to Foxp3+ Treg in MLNs of mice; Increased production of SCFAs; Reshaped gut microbiota.
Bian X. et al. 2019 [34]	Mice with DSS-induced colitis	Live *A. muciniphila*	Improved clinical parameters of colitis (reduced weight loss, colon length shortening, and histopathology scores); Reduced serum and tissue levels of inflammatory cytokines and chemokines.	Enhanced gut barrier function; Reshaped gut microbiota; Increased production of SCFAs.
Kim S. et al. 2021 [35]	Normal mice, germ-free mice, and mice with gut damage induced by radiation and methotrexate	Live *A. muciniphila*	Accelerated proliferation of ISCs in small intestine and colon; Increased differentiation of Paneth cells and goblet cells in the small intestine and colon; *A. muciniphila* pre-treated mice showed less gut damage after radiation and methotrexate administration.	Enhanced Wnt signaling; Increased SCFAs production with SCFAs-mediated Gpr 41/43 activation.
Kump P. et al. 2018 [36]	UC patients refractory to conventional therapy	FMT or antibiotic pre-treatment only	In the FMT group, 59% of patients showed clinical response and 24% a clinical remission, with the stool of donors with a high relative abundance of *A. muciniphila* being more likely to induce remission.	
Fan L. et al. 2021 [37]	*Apc^Min/+^* mice (a spontaneous model of adenoma formation) and nude mice with subcutaneously implanted HCT116 human colon cancer cells or CT26 murine colon cancer cells	Live *A. muciniphila*	Suppressed colonic tumorigenesis in *Apc^Min/+^* mice and suppressed growth of implanted HCT116 or CT26 tumors in nude mice	Enrichment of M1-like macrophages in colon tissue in a TLR2/NLRP3-dependent way.
Chen Z. et al. 2020 [38]	Mice subcutaneously injected with Lewis lung cancer cells	CDDP, CDDP + A. *muciniphila*, and CDDP + antibiotics	Compared with the CDDP group, CDDP combined with *A. muciniphila* reduced tumor growth and volume; Compared with the CDDP group, CDDP combining with *A. muciniphila*, downregulated the levels of ki-67, p53, Fas ligand proteins, and upregulated Fas proteins, increased the levels of proinflammatory cytokines, and reduced the number of Treg lymphocytes in mouse peripheral blood and spleen.	A. *muciniphila* combined with CDDP increased the levels of IFI27l2 and IGFBP7 and thus influenced various pathways, including the cytokine–cytokine receptor interaction, Th17 cell differentiation, FOXO, JAK-STAT, and PI3K-Akt signaling pathways.
Shi L. et al. 2020 [39]	Mice subcutaneously injected with B16F10 melanoma cells and CT26 colon cancer cells	IL-2 or IL-2 + live *A. muciniphila* or IL-2 + Amuc (outer membrane protein)	Compared with the IL-2 group, IL-2 combining with live *A. muciniphila* or Amuc reduced tumor burden and prolonged survival of both murine cancer models; Compared with the IL-2 group, IL-2 combined with live *A. muciniphila* or Amuc increased the production of proinflammatory cytokines and the infiltration of CTLs and reduced the number of Treg lymphocytes in tumor immune microenvironment; Compared with the IL-2 group, IL-2 combined with live *A. muciniphila* or Amuc preserved gut barrier function and gut homeostasis.	Amuc-mediated TLR2 activation.
Routy B. et al. 2018 [40]	Mice with MCA-205 sarcoma and RET melanoma treated with ICIs	Antibiotics, FMT from cancer patients ICIs- responders, FMT from cancer patients ICIs-non responders, live *A. muciniphila*	Antibiotics inhibited the clinical benefit of ICIs in patients with cancer and in mouse models; FMT from cancer patients who responded to ICIs into germ-free or antibiotic-treated mice improved the antitumor effects of ICIs, whereas FMT from nonresponding patients could not; Oral supplementation with *A. muciniphila* after FMT with non-responder feces restored the antitumor efficacy of ICIs.	Supplementation with *A. muciniphila* increased the recruitment of CCR9 + CXCR3 + CD4+ T lymphocytes into mouse tumor beds in an IL-12-dependent way.
Chevalier C. et al. 2015 [41]	Germ-free mice	Cold exposure, cold microbiota transplantation, and live *A. muciniphila*	Cold exposure and cold microbiota transplantation increased the gut absorptive surface and capacity; Administration of cold-suppressed *A. muciniphila* reverted the increased caloric uptake and absorptive surface.	*A. muciniphila* increased the apoptosis levels and reduced the expression of anti-apoptotic and glucose uptake genes upregulated in small intestine epithelial cells during cold exposure or cold microbiota transplantation.
Kim S. et al. 2020 [42]	HFD-fed mice	Live *A. muciniphila*	Reduced serum triglycerides; Reduced liver injury (reduced ALT levels and amelioration of histological fatty changes in liver tissue).	Reduced expression of *SREBP* (regulator of triglycerides synthesis in liver tissue) and IL-6 in liver tissue following administration of *A. muciniphila*; Restored gut homeostasis impaired by HFD.
Org E. et al. 2015 [43]	Obesity-prone mice fed with HF/HS diet	Live and heat-killed *A. muciniphila*	Viable but not heat-killed *A. muciniphila* administration reduced body weight, fat mass, lipid levels, and insulin resistance in HF/HS-fed mice.	Reshaped gut microbiota.
Shin N. et al. 2014 [44]	HFD-fed mice	Metformin, live and heat-killed *A. muciniphila*	Metformin treatment improved the glycemic profile and increased the abundance of *A. muciniphila* in HFD-fed mice; Administration of live but not heat-killed *A. muciniphila* without metformin significantly improved glucose tolerance.	Attenuated adipose tissue inflammation by recruiting Tregs in the VAT.
Zhang L. et al. 2018 [30]	Streptozotocin-induced diabetic rats	Live and pasteurized *A. muciniphila*	Both preparations significantly increased the blood concentration of HDL and decreased the hepatic glycogen, serum PAI-1, TNF-α, lipopolysaccharide, malondialdehyde, and total GLP-1, thereby ameliorating the course of the disease.	
Hänninen A. et al. 2018 [45]	NOD mice	Live *A. muciniphila*	Administration of *A. muciniphila* delayed diabetes development in NOD mice.	Enhanced gut barrier function; Diminished TLR levels, increased Tregs recruitment, and reduced infiltration of mononuclear leukocytes in pancreatic islets; Increased Treg-associated cytokines IL-10 and TGF-β expression in pancreas-draining lymph nodes.
Perraudeau F. et al. 2020 [46]	T2DM patients	WBF-011 (a multistrain probiotic formulation containing inulin, *A. muciniphila, Clostridium beijerinckii, Clostridium butyricum, Bifidobacterium infantis, and Anaerobutyricum hallii*) and WBF-010 (containing inulin, *Clostridium beijerinckii, Clostridium butyricum,* and *Bifidobacterium infantis*)	No issues regarding the safety and tolerability of both products; Improvement in postprandial glucose control in the WBF-011 group (significant decrease in total glucose AUC _0–180 min_ during a glucose-tolerance test); Incremental glucose AUC _0–180 min_ and A1c significantly lower in the WBF-011 group.	Small increase in propionate and butyrate in stool from subjects treated with both WBF-010 and WBF-011 (not statistically significant); Small correlation between changes in A1c and changes in butyrate in subjects administered with WBF-011 (not statistically significant).
Rao Y. et al. 2021 [47]	HFC-diet fed mice	Live *A. muciniphila*	Reduced mouse body weight; Significant MAFLD amelioration (probably related to L-aspartate, which is a potential agent for MAFLD treatment).	Increased lipid oxidation in gut-liver axis; Attenuation of bile acid metabolism dysfunction in obese mice; Enhanced gut barrier function.
Raftar S. et al. 2020 [31]	Quiescent and LPS-activated HSC and HFD-fed mice administered with CCl4	Live and pasteurized *A. muciniphila* + EVs	Reduced expression of fibrosis markers by activated HSC; Amelioration of liver biochemistry; Attenuation of liver histopathological damage; Reduced expression of fibrosis and inflammatory biomarkers in hepatic tissue.	Reduced expression of TLR2 and TLR4 in HSC; Enhanced gut barrier function; Reshaped gut microbiota.
Ou Z. et al. 2020 [48]	APP/PS1 HFD-fed mice	Live *A. muciniphila*	Improvement in fasting blood glucose levels, blood lipid levels, hepatic steatosis, and scapular brown fat whitening; Reduction of Aβ 40–42 levels in the cerebral cortex of APP/PS1 mice and improved cognitive functions.	Reduced gut damage induced by HFD.
Yang Y. et al. 2019 [49]	Early-life HFD-fed mice	Live *A. muciniphila*	Reduction of hippocampal microgliosis and proinflammatory cytokines expression; Restoration of neuronal development and synapse plasticity impaired by early life HFD feeding; Amelioration of defects in learning and memory.	Enhanced gut barrier function; Possible role of TLR4 blockade and gut dysbiosis correction in preventing neuronal defects.
Blacher E. et al. 2019 [50]	ALS-prone Sod1 transgenic mice	Live *A. muciniphila*	Amelioration of ALS symptoms and prolonged animals’ lifespan; Reduced brain atrophy and increased spinal cord cellularity.	Possible *A. muciniphila* NAM-mediated action to ameliorate mitochondrial function, NAD homeostasis, and the clearance of superoxide radicals (functions disrupted in ALS).
Goo N. et al. 2020 [51]	Fmr1 KO mice	FMT	FMT ameliorated autistic-like behaviors and normalized *A. muciniphila* intestinal abundance, which was low at baseline; Reduction of proinflammatory cytokines expression and microglial activation in mice brain.	Restoration of gut barrier integrity.

Abbreviations: HFD—high fat diet; Apoe—Apolipoprotein E; UC—ulcerative colitis; CD—Crohn’ s disease; WMT—washed microbiota transplantation; DSS—dextran sulfate sodium; CD4—Cluster of differentiation 4; Foxp3—forkhead box p3; Treg—T regulators; MLNs—mesenteric lymph nodes; SCFAs—short-chain fatty acids; ISCs—intestinal stem cells; WNT—Wingless-related integration site; Gpr 41/43—G-protein coupled receptor 41/43; FMT—faecal microbiota transplantation; APC—adenomatous polyposis coli; TLR2—Toll-like receptor 2; NLRP3—NLR family pyrin domain containing 3; CDDP—cisplatin; p53—tumor protein 53; Fas—factor-associated suicide; IFI27L2—Interferon Alpha Inducible Protein 27 Like 2; IGFBP7—insulin growth factor binding protein 7; Th17—T helper 17; FOXO—Forkhead box O; JAK-STAT—Janus kinase-Signal transducer and activator of transcription; PI3K-Akt—Phosphatidylinositol-3-Kinase-Protein Kinase B; IL-2—interleukin 2; CTLs—cytotoxic T lymphocytes; Tregs—T regulators; RET—REarranged during Transfection; ICIs—immune checkpoint inhibitors; CCR9—C-C Motif Chemokine Receptor 9; CXCR3—C-X-C Motif Chemokine Receptor 3; IL-12—Interleukin-12; ALT—alanine aminotransferase; SREBP—Sterol regulatory element-binding proteins; IL-6—interleukin-6; HS—high sucrose; VAT—visceral adipose tissue; NOD—non-obese diabetic mice; IL-10—Interleukin-10; TGF-β—Transforming Growth Factor-β; AUC—area under the curve; A1c—glycosylated hemoglobin; HFC—high fat and high cholesterol; MAFLD—metabolic-associated fatty liver disease; APP/PS1—amyloid precursor protein/presenilin 1; Aβ 40–42—Amyloid beta 40–42; ALS—Amyotrophic Lateral Sclerosis; SOD1—superoxide dismutase 1; NAM—Nicotinamide; NAD—Micotinamide adenine dinucleotide; Fmr1 KO—Fragile X Messenger Ribonucleoprotein 1 Knockout.

## 2. *Akkermansia muciniphila* and Inflammatory Bowel Diseases

The gut microbiota plays an undeniable role in the pathogenesis of inflammatory bowel diseases (IBD), and the modulation of the gut microbiota represents one of the most promising challenges in IBD therapy [52,53,54,55,56].

Many case–control studies have documented a significant decrease in the relative abundance of *A. muciniphila* both in ulcerative colitis (UC) and Crohn’s disease (CD) compared to healthy controls [32,57,58,59], with only one study showing an opposite trend in a group of patients affected by CD [60].

As previously mentioned, *A. muciniphila* exerts an anti-inflammatory effect within the intestinal microecology, which was the object of exploratory analyses in the setting of IBD. Among the underlying mechanisms proposed, the production of SCFAs is the most deeply investigated; the production of SCFAs has been demonstrated to protect against colitis by increasing the number of forkhead box P3 (Foxp3+) regulatory T cells in the colon and through the activation of the G-protein coupled receptor 43 (GPR43) expressed by immune cells and colonic epithelium [33,61,62]. Wang et al. observed that the administration of *A. muciniphila* could improve dextran sulfate sodium (DSS)-induced colitis in mice by reducing macrophage and CD8+ cytotoxic T lymphocyte levels in the colon [22], while Bian et al. reported a downregulation of pro-inflammatory cytokines and chemokines [34]. Additionally, the administration of *A. muciniphila* enhances intestinal stem cell proliferation and Paneth and goblet cell differentiation in the small intestine and colon of both healthy mice and mice with gut damage [35].

*A. muciniphila* also restored the mRNA expression of tight junction proteins such as zonulin-1, occludin, and claudin-1 in mouse models of DSS-induced colitis, thereby reducing gut permeability and reshaping the intestinal microbiota, leading it toward eubiosis; these effects are related to the administration of Amuc:2109, a β-acetylaminohexosidase secreted by this microorganism [23].

On the other hand, an increased abundance of *A. muciniphila* was also reported in preclinical models of IBD [63,64,65]. Interestingly, when administered to mice with non-DSS-induced colitis, *A. muciniphila* was associated with symptoms worsening; *A. muciniphila* administration also exacerbated the symptoms of *Salmonella*-*typhimurium*-induced gut inflammation in a mouse model with a background microbiota of eight bacterial species [66], and it was possibly implicated in the worsening of colitis in IL10 −/− mice.

The discrepancy in the effects of this bacterial species could allow for several interpretations, being possibly biased by the different mouse models used; moreover, it can be speculated that the increased abundance of *A. muciniphila* in colitis models could represent a causative factor or rather, a reactive response. When *A. muciniphila* was administered in the IL-10 −/− mice colonized with a simplified human gut microbiota, it did not promote inflammation, suggesting that other environmental conditions could be involved [67,68]. 

Finally, there are few studies on the predictive effect of *Akkermansia* after FMT in patients with IBD. Zhang et al. demonstrated that washed microbiota transplantation (WMT) significantly increased the colonization rate of *Akkermansia* and that there was a positive correlation between the abundance of patient’s and donor’s *Akkermansia* abundance after WMT, speculating its possible role as a predictive factor of WMT efficacy [32]. Similar results were obtained by Kump et al. in treatment-refractory patients with UC; indeed, the stool of donors with a higher bacterial richness and a higher relative abundance of *A. muciniphila*, *Ruminococcaceae*, and *Ruminococcus spp*. were more likely to induce remission in these patients. In particular, *A. muciniphila* was nearly absent in baseline samples but was significantly increased the day after FMT in patients achieving remission [36].

In conclusion, current evidence, although conflicting to some degree, paves the way for a potential role of *A. muciniphila* in IBD treatment [69].

## 3. *Akkermansia muciniphila* and Cancer

Colorectal cancer (CRC) is one of the most common and lethal cancers in the world. Although being overweight and obese, Western dietary habits, smoking, and heavy alcohol consumption are the better-known risk factors for CRC, the intestinal environment has also received widespread attention in this field. It has been demonstrated in humans and in animal models that gut dysbiosis may promote colon carcinogenesis via multiple mechanisms, including the development of chronic inflammation and the production of genotoxins and other microbial products [70,71,72,73].

*A. muciniphila* depletion is also a feature of CRC-associated dysbiosis. In models of colitis-associated CRC (CAC), the administration of pasteurized *A. muciniphila* or Amuc_1100 alone improved symptoms, delayed tumor development, and decreased the number and area of tumor lesions by attenuating DNA damage, cell apoptosis, and abnormal proliferation; the beneficial effects of *A. muciniphila* were associated with the expansion of cytotoxic T-lymphocytes in the colon and mesenteric lymph nodes and with the modulation of macrophages subpopulations, thus explaining how *A. muciniphila* influences inflammation-associated tumorigenesis [22].

Another study further confirmed that the abundance of *A. muciniphila* is significantly reduced in humans with CRC and that its supplementation can inhibit colonic tumorigenesis in ApcMin/+ mice via the expansion of M1-like macrophages in colonic tissue. Tumor-associated macrophages (TAMs) can assume a pro-inflammatory polarization (M1) or an anti-inflammatory polarization (M2), with only the former helping to suppress cancer cells. This effect is mediated by the interaction between *A. muciniphila* and the TLR2 expressed by macrophages, with the subsequent activation of the NOD-like receptor family pyrin domain containing 3 (NLRP3) intracellular pathway [37,74,75].

Apart from its immunomodulatory effects, *A. muciniphila* can also directly interfere with colon carcinogenesis through the production of Amuc_1434, an enzyme that can degrade Mucin2, the main component of the intestinal mucus layer, which is highly expressed in mucinous CRC. Amuc_1434 showed a protective effect on tumor protein 53 (p53) expression in vitro, resulting in the blockade of the G0/G1 cell cycle phase and the promotion of CRC cells apoptosis [24,76].

Conversely, it was observed that *A. muciniphila* abundance was heavily increased in two different cohorts of patients affected by CRC, as well as in a cohort of patients with esophageal and gastric cancers compared with healthy controls [77,78]. However, according to Weil et al., this observation can be related to an increased substrate availability rather than to a detrimental role of this bacterium, considering the overexpression of MUC1 and MUC5AC in CRCs [78].

Besides CRC, *A. muciniphila* was found to be more abundant in patients with non-small-cell lung cancer (NSCLC) and to gradually decrease during the progression from cirrhosis to hepatocellular carcinoma [79,80,81].

In recent years, gut microbiota modulation applied to cancer therapy is certainly a topic of growing interest in either treatment efficacy or tolerability [38,82,83].

There are some data regarding the possible role of *A. muciniphila* in both conventional and targeted anticancer therapy. For instance, *A. muciniphila* could improve the antitumor effect of cisplatin; in mouse models of lung cancer, the administration of *A. muciniphila* in combination with cis-diamminedichloroplatinum (CDDP) was associated with reduced tumor growth, the downregulation of ki-67, p53, factor-associated suicide (Fas) ligand proteins, and the upregulation of Fas proteins [38]. Moreover, the administration of *A. muciniphila* in this setting positively affected the production of cytokines toward a pro-inflammatory profile, suppressing the development of T-reg lymphocytes. This suggests that *A. muciniphila* could modulate the immune microenvironment toward an inflammatory response, counteracting tumor immune escape. *A. muciniphila* has been proven to enhance the antitumor efficacy of interleukin (IL)-2; in murine models of melanoma and CRC, the combined administration of IL-2 and *A. muciniphila* reduced the tumor burden and improved survival compared with IL-2 treatment alone, primarily by stimulating the response of CD4+ and CD8+ T cells against cancer cells and by decreasing the number and the activity of T-regs. These beneficial effects were at least partially mediated by TLR2 signaling activated by a specific membrane protein [39]. The current literature also shows a peculiar interplay between *A. muciniphila* and abiraterone acetate (AA), an inhibitor of androgen biosynthesis for the treatment of prostate cancer (PCa) refractory to androgen deprivation therapy (ADT). In a cohort of PCa patients, AA administration increased the abundance of *A. muciniphila*. This was independent of immunological modulation and possibly resulted from the interaction between the conjugated acetate portion of AA and *A. muciniphila* [84,85,86]. Unfortunately, the authors did not explore the contribution of *A. muciniphila* to the efficacy of AA. In a later study, the intravenous administration of *A. muciniphila*-derived extracellular vesicles in PCa-bearing, immune-competent mice operated as an immune modulator; it was associated with the increased activation of CD8+ T cells and tumor-killing M1 macrophages, resulting in a reduced tumor mass [25].

To further clarify the role of *Akkermansia* in cancer immunotherapy, Xu et al. evaluated the effects of the modulation of the gut microbiome on the response to immune checkpoint inhibitors (ICIs). In CRC mouse models, the exposure to several broad-spectrum antibiotics interfered with the efficacy of programmed cell death protein 1 (PD-1) antibodies, depending on the type of antibiotic and the resulting changes in the gut microbiota composition. In particular, the authors conducted a metagenomic analysis to assess the correlation between specific bacterial taxa and metabolic and immunologic changes. *A. muciniphila* was found to be enriched in the vancomycin-treated group and associated with a better outcome; according to the authors, *Akkermansia* could preserve the efficacy of anti-PD-1 therapy by modulating the metabolism of glycerophospholipids, which influence the expression of immune-related cytokines IFN-γ and IL-2 in the tumor microenvironment [87]. Other published studies in patients with hepatocellular carcinoma and melanoma highlighted the contribution of the gut microbiome to the response to immunotherapy, and *A. muciniphila* emerged as a key element associated with treatment efficacy [40,88,89,90,91].

Finally, in patients with NSCLC and renal cell carcinoma (RCC) undergoing immunotherapy with ICIs, FMT from treatment responders to germ-free mice resulted in increased efficacy of immunotherapy. *A. muciniphila* was found to be more abundant and associated with treatment response, and the oral administration of *A. muciniphila* improved PD-1 blockade effectiveness, once again through the modulation of the immune response, specifically by promoting the recruitment of CD4+ T cells [40].

## 4. *Akkermansia muciniphila* and Metabolic Diseases

The prevalence of obesity is increasing worldwide, with growing concerns about the healthcare burden associated with its complications. Obesity is a risk factor for cardiovascular disease, diabetes mellitus, chronic kidney disease, several cancers, and musculoskeletal disorders [92,93]. Current evidence shows that obesity is associated with specific changes in the gut microbiota, and *A. muciniphila* is negatively correlated with body weight in both humans and in mouse models [94,95,96,97,98,99,100,101]. However, to date, the mechanisms by which *A. muciniphila* can modulate body weight and metabolism have not been completely clarified. *A. muciniphila* can increase the secretion of glucagon-like peptide-1 (GLP-1), a hormone able to increase insulin secretion by reducing postprandial glucose spikes, to reduce the expression of glucose and fructose transporters in the jejunum, with a consequent reduction in carbohydrate absorption, and to modulate the expression of proteins involved in adipose cell differentiation, thus influencing body weight and composition [41,102,103]. These beneficial effects of the host–microbe interaction could be also mediated by the endocannabinoid system, which stimulates the secretion of metabolically active molecules such as glucagon-like peptides [20,104]. Moreover, it has been suggested that the protective effect of *A. muciniphila* on the intestinal barrier can reduce systemic inflammation, resulting in an overall improved metabolism [13,26,42].

Since there is an inverse correlation between the abundance of *A. muciniphila* and metabolic diseases, its therapeutic potential has been widely explored in several studies. Plovier et al. demonstrated that in a murine model of obesity induced by high-fat diet (HFD), the administration of pasteurized *A. muciniphila* reduced body weight gain and the accumulation of white adipose tissue while improving insulin resistance and dyslipidemia; these effects were ascribed to the interaction between Amuc_1100 and TLR2. The latter has been implicated in the pathogenesis of metabolic disorders such as obesity and type 2 diabetes mellitus [14,105,106]. Another preclinical study reported a similar result, demonstrating an association with the restoration of the integrity of the intestinal barrier damaged by HFD, and that *A. muciniphila* administration in mice could prevent liver steatosis and improve hepatic function [27].

Depommier et al. confirmed that the oral administration of pasteurized *A. muciniphila* mitigates diet-induced obesity due to an increased fecal energy excretion, which is likely dependent on a reduction of carbohydrate absorption. This study also evidenced a reduced expression of perilipin 2, a protein associated with lipid storage, in brown and white adipose tissues, induced by the administration of *A. muciniphila* [28].

As opposed to the previously mentioned studies, *A. muciniphila* treatment reversed HFD-induced metabolic disorders in mice by increasing intestinal levels of endocannabinoids, which control inflammation, the integrity of the gut barrier, and gut peptide secretion. However, this only occurred after the administration of the viable microorganism, as the heat-killed forms did not achieve the same positive results [5]. Another study obtained an amelioration of metabolic parameters with either viable or heat-killed *A. muciniphila* [43].

A remarkable effect on glucose metabolism has been also highlighted, as treatment with metformin increases the abundance of *A. muciniphila* in HFD-fed mice by improving the glycemic profile. However, oral administration of *A. muciniphila* alone also significantly enhanced glucose tolerance and attenuated adipose tissue inflammation by locally recruiting T-regs [44]. Even in T2DM patients, metformin increased the levels of *A. muciniphila* in feces, thus suggesting another possible mechanism of action of this drug. Furthermore, patients with T2DM who did not achieve optimal glycemic control using metformin or other hypoglycemic agents had a lower relative abundance of *A. muciniphila* compared to responders [44,107,108,109].

Considering the promising effects of *A. muciniphila* on metabolic disorders in animal studies, some clinical trials have been conducted. Two of them have already been completed and published, with one specifically regarding patients with metabolic syndrome [102]. Depommier et al. conducted a randomized, controlled, double-blind clinical trial enrolling a total of 32 participants with a diagnosis of metabolic syndrome to assess the beneficial effects of *A. muciniphila*. After a 3-month treatment with pasteurized *A. muciniphila,* the authors observed a significant improvement in insulin resistance and plasma cholesterol levels, with a slight decrease in weight, fat mass, and hip circumference, and a good safety and tolerability profile [29]. Two other ongoing clinical trials are currently assessing the effects of *A. muciniphila* administration in obesity and type 2 diabetes mellitus (T2DM) (NCT: NCT04797442) and on insulin resistance in healthy individuals with dysglycemia (NCT: NCT05114018) [102].

*Akkermansia* is also a promising predictive factor of the success of dietary interventions; indeed, it was shown that the baseline abundance of *A. muciniphila* positively correlated with an improvement in blood glucose and lipid levels and body fat distribution following the dietary intervention [85]. Conversely, several dietary interventions have been tested in human and animal studies to enhance the intestinal abundance of *A. muciniphila*. Various types of dietary fibers, calorie restriction, and polyphenol-rich foods such as wild blueberry polyphenolic extract, cranberry extract, and grape polyphenols, appear to be the most effective [85,103,110,111,112,113]. Moreover, the administration of prebiotics such as fructooligosaccharides and inulin has been reported to increase *A. muciniphila* abundance in mice [114,115]. In contrast, *A. muciniphila* abundance has been negatively affected by energy-rich diets, such as a high-fat diet (HFD), high-fat, high-sugar diet (HFHS), or high-fat, high-cholesterol diet (HFHC) [116]; some of these studies also reported an improvement in metabolic parameters after nutritional interventions that was found to correlate with an increase in *A. muciniphila* abundance [111,117].

Despite the differences in etiopathogenesis, both T1DM and T2DM couple with gut dysbiosis [118,119,120,121]. Although there is very limited evidence reporting the correlation between *A. muciniphila* and T2DM, most metagenomic studies outlined an inverse correlation between its abundance and glycemia, also in a preclinical phase of the disease [98,122,123,124].

*A. muciniphila* has also been used for the treatment or prevention of diabetes in mouse models. In experimental models of diabetes induced by streptozotocin, the administration of *A. muciniphila* improved liver function, reduced glucotoxicity and lipotoxicity, alleviated oxidative stress, and suppressed inflammation [30]. In non-obese diabetic (NOD) mice, vancomycin treatment increased *A. muciniphila* intestinal abundance and at the same time, reduced the incidence of T1DM, thus suggesting a protective role of *A. muciniphila* against T1DM development [125]. Furthermore, NOD mouse colonies with a lower incidence of T1DM exhibited a greater abundance of *A. muciniphila*, and its administration to animals with a high incidence of T1DM was able to delay disease development [45]. Beyond the promising effects of *A. muciniphila* on diabetes in animal models, consistent findings have also emerged in humans. Perraudeau et al. showed that T2DM patients treated with WBF-011 (a product containing inulin, *Akkermansia muciniphila*, *Clostridium beijerinckii*, *Clostridium butyricum*, *Bifidobacterium infantis*, and *Anaerobutyricum hallii*) for 12 weeks significantly improved postprandial glucose control and glycated hemoglobin compared with a placebo, with no issues about safety or tolerability. The probiotic strains in this formulation interacted mutually to form butyrate, which further stimulates GLP-1 release, thus explaining the results [46,103].

Recently, the gut microbiome has been gaining extensive attention for the treatment of metabolic-associated fatty liver disease (MAFLD) as well [103,126]. MAFLD (previously known as non-alcoholic fatty liver disease, NAFLD) is a metabolic syndrome characterized by lipid accumulation in the liver and subsequent oxidative stress, inflammation, apoptosis, and various degrees of fibrosis [127,128,129]. Obese mice treated with *A. muciniphila* reported the regression of MAFLD in association with the enhancement of lipid oxidation in the liver and by strengthening the gut mucosal barrier [47]. Similarly, Kim et al. observed that the oral administration of *A. muciniphila* significantly improved hepatic steatosis in obese mice by reducing the synthesis of triglycerides in the liver; additionally, *A. muciniphila* administration improved gut integrity and mitigated liver inflammation in these models [42]. Another study aimed to assess the beneficial effects of *A. muciniphila* treatment on the prevention of liver fibrosis showed that it significantly correlated with a reduction in both hepatic stellate cell (HSC) activation and fibrosis stage in mice fed with HFD after the administration of carbon tetrachloride (CCl4) [31].

There are currently two other ongoing clinical trials in the recruitment phase that are assessing the effects of *A. muciniphila* administration in patients affected by obesity and T2DM (NCT: NCT04797442) and in healthy individuals with dysglycemia (NCT: NCT05114018) [102].

## 5. *Akkermansia muciniphila* and Atherosclerosis

A significant association between atherosclerosis and specific components of the gut microbiota has been reported by several human studies, prompting the reconsideration this condition as a microbiota-associated disease [130,131,132,133]. Given this evidence, some authors tried to address the exact role of *A. muciniphila* in this condition; in a study by Li et al., Apolipoprotein E (ApoE)−/− mice prone to the atherosclerotic disease on a normal or Western diet were treated with live *A. muciniphila*. The treatment significantly reduced the dimensions of the atherosclerotic lesions of the aorta induced by the Western diet and reduced local and systemic inflammation as well. Changes were associated with a marked attenuation in metabolic endotoxemia and gut permeability, suggesting that *A. muciniphila* could attenuate atherosclerotic lesions through the restoration of gut barrier function [21]. On the contrary, *A. muciniphila* has also been associated with the production of trimethylamine *n*-oxide (TMAO), a metabolite of L-carnitine that was reported to accelerate atherosclerosis through the impairment of the reverse cholesterol transport and the shift of the phenotype of artery wall macrophages [134]; however, the authors did not investigate whether *A. muciniphila* was associated with enhanced atherogenesis [135,136].

## 6. *Akkermansia muciniphila* and Neurological Diseases

### 6.1. Parkinson’s Disease

Colonic inflammation and increased gut permeability have been shown to be associated with Parkinson’s disease (PD) [137,138,139]. It has been demonstrated in animal models that neuronal injury and the aggregation of the protein α-synuclein (aSyn), which is responsible for neuronal loss in the substantia nigra in patients with PD, can be triggered by toxins or the translocation of bacterial proteins or can even start in the enteric nervous system and spread to the central nervous system [137,138,139,140,141]. For these reasons, there is a growing interest in characterizing changes in the intestinal bacterial populations associated with PD. Several studies reported an increased abundance of *A. muciniphila* in the fecal samples of patients with PD compared with healthy donors [142,143,144,145,146,147,148,149], and these observations were confirmed by a recent meta-analysis which also took into account possible confounding factors, such as constipation, BMI, sex, age, and drug intake [150]. The hypothesis functionally related to this observation is that *A. muciniphila*, being involved in mucus turnover, can increase gut permeability in these patients via the production of hydrogen sulfide, altering the integrity of the intestinal barrier and enhancing the absorption of bacterial toxins [151]. Further studies are needed to confirm these mechanisms.

### 6.2. Multiple Sclerosis

Multiple sclerosis (MS) is a demyelinating disease of the central nervous system whose etiology is still unknown [152,153]. In mouse models, commensal bacteria can trigger a spontaneous form of experimental autoimmune encephalomyelitis (EAE) after exposure to myelin oligodendrocyte glycoprotein [154,155]; nevertheless, germ-free mice prone to brain autoimmunity who received FMT from patients with MS showed more severe symptoms of EAE than mice who received FMT from healthy controls [156]. This suggests the role of the gut microbiota in the pathogenesis of MS.

Several studies and systematic reviews report an overabundance of *A. muciniphila* in the gut microbiota of patients affected by MS, either in the untreated ones or in those receiving various disease-modifying therapies [156,157,158,159,160,161]. These data were also confirmed by comparing patients with their genetically unrelated household healthy controls, thus minimizing the effects of diet and environmental factors on the gut microbiome composition [162].

In a particular observational study comparing the gut microbiota composition of 34 monozygotic twin pairs discordant for MS, an increased abundance of *A. muciniphila* was reported in the untreated twins affected by MS compared with either the healthy twins or those affected by MS who were receiving treatment; FMT from twins with MS induced a significantly higher incidence of disease in transgenic mouse models of spontaneous brain autoimmunity, with IL-10 being involved in preventing disease development [157].

Notably, cannabinoids, which are used to counteract muscle spasticity, reduced the abundance of *A. muciniphila* in stools, systemic inflammation, and LPS levels in a mouse brain, improving clinical conditions. Antibiotic treatment resulted in reduced disease severity in EAE mice, while FMT from EAE mice exposed to cannabinoids to those treated with antibiotics resulted in an even better outcome, confirming the critical role of cannabinoids play in attenuating EAE through the modulation of the gut microbiome [163].

To explain these findings, it was suggested that *A. muciniphila* might be involved in the activation/expansion of autoreactive memory CD4+ T cells or the production of cross-reactive antibodies via molecular mimicry in predisposed individuals [164,165,166]. Otherwise, *A. muciniphila* may induce dysfunction of the intestinal barrier under certain circumstances, e.g., in case of nutritional deficiencies or through interaction with other bacterial species [167,168,169,170,171].

However, there is also evidence regarding the protective role of *A. muciniphila* in central nervous system autoimmunity. Some authors hypothesized that the increased abundance of *A. muciniphila* in the early stage of the disease could be a compensatory mechanism aimed at preventing disease progression [172,173,174]. This makes it challenging to conclude the exact role of *A. muciniphila* in MS pathogenesis, even in animal models.

### 6.3. Alzheimer’s Disease

The gut microbiota can release a significant quantity of amyloids and lipopolysaccharides, which might influence signaling pathways and lead to the production of proinflammatory cytokines related to the pathogenesis of Alzheimer’s disease (AD) [175,176,177]. Colonization of germ-free amyloid β precursor protein (APP) transgenic mice with gut microbiota from conventionally raised APP transgenic mice increased cerebral levels of the amyloid β-protein (Aβ), while colonization with the gut microbiota from wild-type mice was less effective in inducing these changes, indicating a role of the gut microbiota in AD development [178].

Significant depletion of *A. muciniphila* was found in fecal samples from APP transgenic mice prone to developing AD when compared to wild-type mice and was negatively correlated with the amount of the pathogenic Aβ 42 in the brain.

A later study confirmed that the abundance of *A. muciniphila* decreased in mouse models of AD alongside a reduction in the number of colonic mucus cells and an increase in serum levels of diamine oxidase (DAO), which reflects an impairment of intestinal barrier function; the administration of *A. muciniphila* effectively reduced serum DAO, reverted the loss of colonic mucus cells, promoted the reduction of pathogenic Aβ 40–42 levels in the cerebral cortex, and improved cognitive abilities in AD mouse models [48].

Recent evidence indicated that metabolic disorders, including obesity and insulin resistance, are risk factors for cognitive impairment and dementia [176,179,180,181]. The linkage between metabolic impairment and the course of AD was also explored. Early-life feeding with a HFD lead to microgliosis in mice and the expression of proinflammatory cytokines in the hippocampus: this resulted in impairment in neuronal development, spatial learning, and memory, which was associated with an alteration of the gut microbiota composition, again characterized by the depletion of *A. muciniphila* [49]. Oral supplementation with *A. muciniphila* improved gut permeability and reduced hippocampal microgliosis and the expression of proinflammatory cytokines, restoring neuronal development and defects in learning and memory.

Human studies on the topic are lacking. It is worth mentioning that it has been observed that a modified Mediterranean–ketogenic diet could ameliorate cerebrospinal fluid markers of AD in patients with mild cognitive impairment and is associated with an increased fecal abundance of *A. muciniphila* [182].

### 6.4. Amyotrophic Lateral Sclerosis

Environmental factors have been proposed as modulators of the clinical course of amyotrophic lateral sclerosis (ALS), in particular by influencing the composition of circulating low-molecular-mass metabolites that originate from the gastrointestinal tract and enter the blood–brain barrier to modulate metabolic, transcriptional, and epigenetic programs in neurons and other resident cells [183,184]. The gut microbiome is a potential source of these bioactive molecules and has been linked to ALS in animal and human studies [50,185,186,187]. Blacher et al. showed that *A. muciniphila* is less abundant in ALS-prone superoxide dismutase transgenic mice and that its administration ameliorates disease symptoms, prolongs the lifespan of these animals, reduces brain atrophy, and increases spinal cord cellularity. Supplemented mice accumulated nicotinamide (NAM) in the central nervous system, and the systemic supplementation of NAM itself improved motor symptoms and gene expression patterns in the spinal cord. Therefore, *A. muciniphila* could act via NAM to ameliorate mitochondrial function, NAM adenine dinucleotide (NAD) homeostasis, and the clearance of superoxide radicals, functions known to be impaired in ALS [50].

However, it should be taken into account that gut dysbiosis could be a consequence of altered feeding or rather of the use of food supplements or exposure to antibiotics, which are frequent in these patients [188]. Therefore, strong evidence is still required to confirm the role of gut microbiota in ALS.

### 6.5. Autism Spectrum Disorder

Autism spectrum disorder (ASD) is a neuro-developmental disorder of children, characterized by a specific behavioral phenotype of impaired social communication and stereotypic behavior. Once considered uncommon, it is now recognized to involve 1% of the population worldwide [189].

Although still uncertain, the etiology of autism spectrum disorder (ASD) is assumed to reside in the interplay of genetic, epigenetic, and environmental factors, leading to an imbalance in neurotransmitters, dysfunctional neuronal pathways, and abnormal synaptogenesis and neuronal connectivity. Strong associations were found with several genetic and metabolic disorders [190]; gastrointestinal disturbances are also very common in patients with ASD, with significant differences in the gut microbiota profile compared with non-affected children [191,192,193,194].

A statistically significant decreased abundance of *A. muciniphila* in patients with ASD and their siblings compared to healthy controls was reported [195]. Notably, the modulation of the gut microbiota with antibiotics and probiotics improves behavior and bowel health outcomes [196,197].

In a recent study, FMT from normal mice to a mouse model of fragile X syndrome, which is associated with ASD, ameliorated autistic-like behavior, especially memory deficit and social withdrawal, and normalized *A. muciniphila* intestinal abundance, which was low at baseline. The increase in *A. muciniphila* was correlated with a reduction in TNFα and Iba1 (a marker of microglial activation) and with an increased expression of MUC2, suggesting that *A. muciniphila* can improve the course of the disease by restoring intestinal barrier integrity through the stimulation of mucin production and release [51].

## 7. Conclusions

*A. muciniphila* has been gaining increasing attention in recent years, with consistent evidence suggesting its crucial role in the homeostasis of the gut ecosystem and beyond. Indeed, the production of small metabolites and mediators, the influence on microbial diversity and the preservation of the gut barrier integrity promoted by *A. muciniphila* have been proven to exert a beneficial effect not only on the gut but also on a series of diseases involving the metabolic, cardiovascular, neurological, and even oncological fields. The administration of *A. muciniphila* could exert a significant effect on metabolic syndrome and T2DM, being able to affect significantly the course of the disease, thus representing an intriguing adjuvant intervention. On the other hand, data regarding neurodegenerative diseases and anticancer therapy are still lacking and inconclusive, partially due to a series of confounding factors persistently associated with these cohorts of patients. However, further studies are needed to address the precise role of this bacterial species in these topics, specifically in analyzing the administration of live, pasteurized, and single components of *A. muciniphila* in order to make the most of its promising features.

## Data Availability

Not applicable.
